# Cyclic Lipodepsipeptides From *Pseudomonas* spp. – Biological Swiss-Army Knives

**DOI:** 10.3389/fmicb.2018.01867

**Published:** 2018-08-14

**Authors:** Niels Geudens, José C. Martins

**Affiliations:** NMR and Structure Analysis Unit, Department of Organic and Macromolecular Chemistry, Ghent University, Ghent, Belgium

**Keywords:** amphiphile, lipopeptides, *Pseudomonas*, biological activities, metabolite

## Abstract

Cyclic lipodepsipeptides produced by *Pseudomonas* spp. (Ps-CLPs) are biosurfactants that constitute a diverse class of versatile bioactive natural compounds with promising application potential. While chemically diverse, they obey a common structural blue-print, allowing the definition of 14 distinct groups with multiple structurally homologous members. In addition to antibacterial and antifungal properties the reported activity profile of Ps-CLPs includes their effect on bacterial motility, biofilm formation, induced defense responses in plants, their insecticidal activity and anti-proliferation effects on human cancer cell-lines. To further validate their status of potential bioactive substances, we assessed the results of 775 biological tests on 51 Ps-CLPs available from literature. From this, a fragmented view emerges. Taken as a group, Ps-CLPs present a broad activity profile. However, reports on individual Ps-CLPs are often much more limited in the scope of organisms that are challenged or activities that are explored. As a result, our analysis shows that the available data is currently too sparse to allow biological function to be correlated to a particular group of Ps-CLPs. Consequently, certain generalizations that appear in literature with respect to the biological activities of Ps-CLPs should be nuanced. This notwithstanding, the data for the two most extensively studied Ps-CLPs does indicate they can display activities against various biological targets. As the discovery of novel Ps-CLPs accelerates, current challenges to complete and maintain a useful overview of biological activity are discussed.

## Introduction

Cyclic lipodepsipeptides (CLPs) are secondary metabolites with a broad array of biological functions. They are produced by non-ribosomal peptide synthetases (NRPSs) in bacteria, mostly *Pseudomonas, Bacillus*, and *Streptomyces* spp. (Raaijmakers et al., 2010). Interest in CLPs is primarily driven by two projected applications: combating multi-drug resistant pathogens in a clinical setting and use as agents for plant biocontrol and biostimulation in agriculture. The first is illustrated by daptomycin (Cubicin^®^), a CLP produced by *Streptomyces* spp. which is FDA-approved for the treatment of complicated skin and soft-tissue infections caused by Gram-positive bacteria (Arbeit et al., 2004). The second one is exemplified by the fact that several CLP-producing bacteria, including *Pseudomonas* spp., are registered with the United States Environmental Protection Agency (EPA) for biocontrol of plant diseases linked to CLP metabolite production (Olorunleke et al., 2015b) illustrating their potential in agriculture. However, the functional role and activity profile of CLPs is considerably broader (Raaijmakers et al., 2010) and continues to expand, notably with the more recent discovery of insecticidal and anti-carcinogenic activities (Saini et al., 2008; Jang et al., 2013; Cautain et al., 2015). Thus this class of compounds appears as molecular “Swiss-Army” knives, capable of demonstrating a variety of biological and functional effects for the producing organism.

From a chemical point of view, CLPs isolated from *Pseudomonas* spp. show a much larger structural diversity than *Bacillus* spp. CLPs where only three structurally quite similar groups have been described (surfactins, iturins, and fengycins) (Raaijmakers et al., 2010; Meena and Kanwar, 2015). The variations in overall sequence length, macrocycle length, amino acid composition, and stereochemistry should in principle offer possibilities to relate their observed function to (primary) structure. Once available such structure-function understanding should promise a better appreciation of these metabolic products and their underlying biological mechanisms.

Efforts have already been made to catalog CLP primary structures and their producing organisms, such as the NORINE database (Caboche et al., 2008). However, the access to biological activity data is less well, if at all, developed. Review papers of relevant literature, such as that by Raaijmakers et al. (2010) for *Pseudomonas* CLPs, currently remain the single most important source of information in this respect. As a first step toward a more structured overview, we review the biological activity of *Pseudomonas* spp. CLPs to provide the research community with a recent body of information and facilitate access to the primary literature on Ps-CLPs using an overview organized in tabular format. We specifically focus on the biological activity of CLPs originating from *Pseudomonas* spp. (Ps-CLPs) as other reviews are available that cover those of other organisms in considerable detail (Perez-Garcia et al., 2011; Cochrane and Vederas, 2016; Kleijn and Martin, 2017). Moreover, we only considered the data from tests that were performed using purified Ps-CLPs, excluding those using medium or cell extracts. The collected biological data, consisting of 775 reported activities for 51 Ps-CLPs is used to analyze to what extent the comparison of biological activities can already be performed and examine certain generalizations that have been made in the past. Both are preceded by a short overview and description of Ps-CLPs, while results pertaining to the mechanism of action are mentioned for completeness but not exhaustively covered. Rather, the focus lies on extracting recommendations as to possible steps to be made in order to improve prospect for structure-function analysis and future compound application and development.

## *Pseudomonas* spp. Produce A Large Variety of CLPs

**FIGURE 1:**
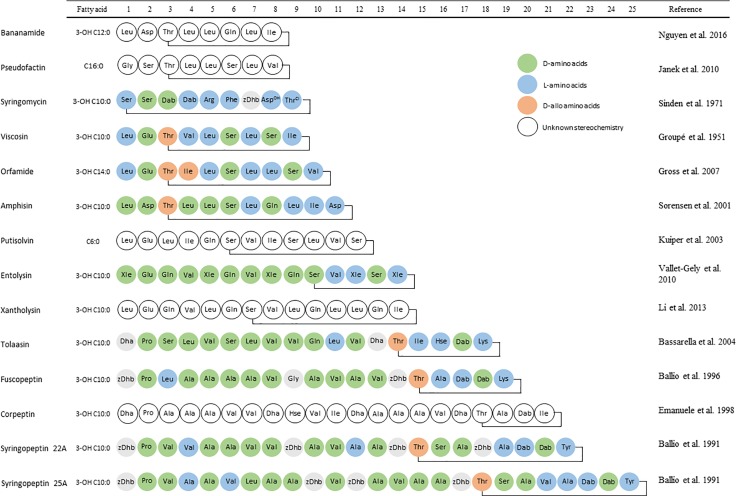
Amino acid sequences of the parent compounds used as basis for Ps-CLP group names. L-amino acids are indicated in blue, while D- and D-allo amino acids are indicated in green and orange, respectively. Amino acids with unknown stereochemistry are indicated in white.

*Pseudomonas* spp. are cosmopolitan organisms found in a multitude of habitats including soil, organic matter, water, the rhizosphere, plants and animals, including mammals. The vast array of metabolites produced includes CLPs of which close to 100 have been individually documented to varying extent. Like for *Bacillus* CLPs, one defining property that Ps-CLPs have in common, due to the presence of a fatty acid tail, is their surface tension lowering properties, designating them as biosurfactants (Fechtner et al., 2011). As more Ps-CLPs were isolated, specific families or “groups” were defined with chemically similar Ps-CLPs and named for a particular prototype Ps-CLP as shown in **Figure 1**. These include the viscosin (Groupé et al., 1951), orfamide (Gross et al., 2007; Ma et al., 2016a), amphisin (Sorensen et al., 2001), syringomycin (Segre et al., 1989), syringopeptin (Ballio et al., 1991), and tolaasin groups (Rainey et al., 1991; Bassarello et al., 2004). New CLPs are continuously found in numerous environments, and can be assigned to an existing group (D’Aes et al., 2014; Weisshoff et al., 2014; Johnston et al., 2015; Michelsen et al., 2015a; Zachow et al., 2015; Ma et al., 2016a; Gotze et al., 2017) or constitute a new group, such as the recently discovered bananamides, (Nguyen et al., 2016) and xantholysins (Li et al., 2013). Finally, a variety of Ps-CLP have not yet formally been classified as a group (**Figure 1**) but can be treated as such, if the oligopeptide length is used as criterion. These include the entolysins (Vallet-Gely et al., 2010), putisolvins (Kuiper et al., 2003), pseudofactins (Janek et al., 2010), syringopeptins (Ballio et al., 1991), corpeptin (Emanuele et al., 1998), and fuscopeptins (Ballio et al., 1996). With about 100 unique Ps-CLPs distributed over 14 groups characterized so far, the chance for rediscovery is obviously increasing, yet the expectation is that not all Ps-CLP groups have been uncovered so far (Nguyen et al., 2016). With so many unique Ps-CLP described and to avoid confusion, we will indicate the individual CLP group members with the first letter of the group name between brackets, unless the CLP name is the same as the group name. For example, WLIP, a CLP of the viscosin group will be designated as WLIP-(V), while tensin, a CLP of the amphisin group will be denominated as tensin-(A).

Cyclic lipodepsipeptides are produced by NRPSs, protein complexes consisting of large multi-domain modules. Current understanding of the processes involved is well developed, as collected in the excellent review from Süssmuth and Mainz (2017). Additionally, Roongsawang et al. (2010) reported a clear summary on the genomics of the NRPS of *Pseudomonas* spp. per lipopeptide-producer. Often, a single biosynthetic NRPS cluster produces more than one CLP due to an assumed flexibility of some adenylation domains that are responsible for amino acid recognition. This results in the production of so-called minors that differ in the identity of a single amino acid, in addition to the major compound. It is unclear whether the minors represent a functional role.

Most likely, CLP production by bacteria is most relevant in competitive interactions with other coexisting bacteria, fungi, and oomycetes as well as during interactions with protozoan predators thus explaining their antimicrobial nature. In contrast, other activities appear to be unintentional but potentially beneficial side-effects. Indeed, it seems unlikely that in their natural habitats CLP-producing bacteria are affected by viruses (Raaijmakers et al., 2010). A similar reasoning applies for the anticancer activities that were reported more recently for several CLPs. In what follows, we chose to review the biological activity of Ps-CLPs according to the type of organism that was used to assess a possible biological effect, rather than by group of CLPs. However, a table correlating biological activity vs. CLP collected in the process also allows for this alternative view (**Supplementary Material**).

### Activity Against Bacteria

Cyclic lipodepsipeptides display clear antagonistic activities against Gram-positive bacteria. Syringopeptin 22A, syringopeptin 25A, corpeptin-(SP), tolaasin, and WLIP-(V) all inhibit growth of *Bacillus megaterium* at similar concentrations (Emanuele et al., 1998; Grgurina et al., 2002; Bassarello et al., 2004; Rokni-Zadeh et al., 2012) while conflicting activities have been reported for syringomycin E and syringotoxin-(SM) (Lavermicocca et al., 1997; Scaloni et al., 2004). However, early reports of the biological activity of syringomycin Ps-CLPs were possibly performed with impure preparations that likely contained syringopeptins. Generally, syringopeptins have a higher activity than syringomycins and syringotoxins-(SM) against *Rhodococcus* and *Micrococcus* species (Lavermicocca et al., 1997).

The CLPs of the structurally quite distinct viscosin and syringopeptin group possess an antagonistic activity against several *Staphylococcus aureus* subspecies, including methicillin-susceptible and methicillin-resistant strains. Only WLIP-(V) (D-aIle4, D-Leu5) and massetolide A-(V) (D-Val4, L-Leu5) lacked activity against all of the tested *S. aureus* strains (Gerard et al., 1997; Grgurina et al., 2005; Andolfi et al., 2008; Reybroeck et al., 2014; Geudens et al., 2017).

**FIGURE 2:**
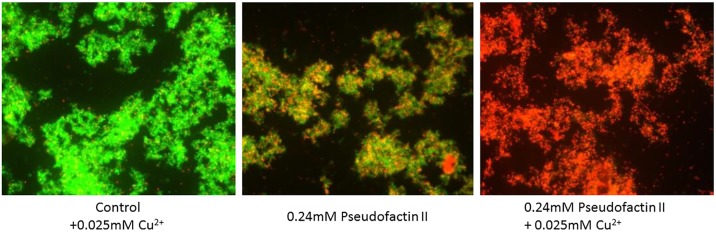
Fluorescence microscopy of antagonistic activity of pseudofactin II against *Staphylococcus epidermidis* KCTC 1917 bacterial cells. The cells are labeled with a bacterial-viability dye, in which viable cells are green and dead cells are red. Figure reprinted from Janek et al. (2016) with permission.

The role of metal ions, mostly Ca^2+^, is often considered when assessing antimicrobial activity. For instance, the CLP daptomycin from *Streptomyces* spp. requires the presence of calcium ions in order to exert its biological function (Taylor and Palmer, 2016). More specifically, in complex with Ca^2+^, daptomycin forms pore-like oligomers on the bacterial cytoplasmic membrane, leading to leakage of intracellular ions, followed by rapid cell death. However, the antibacterial properties of massetolide A-(V), orfamide A, arthrofactin-(A), syringomycin E, and entolysin B are found to be independent of the presence of calcium (Reder-Christ et al., 2012). The antibacterial activity of pseudofactin II was reported to increase in the presence of physiological concentrations of various metal ions including Ca^2+^, Mg^2+^, Zn^2+^, and Cu^2+^ (**Figure 2**), but the underlying working mechanism remains unclear (Janek et al., 2016).

The vast majority of Ps-CLPs is reported not to show antagonistic activities against Gram-negative bacteria (Lavermicocca et al., 1997; Grgurina et al., 2005; Lo Cantore et al., 2006; Andolfi et al., 2008; Sinnaeve et al., 2009a,b; Reybroeck et al., 2014). As a result, *Pseudomonas* CLPs tend to be considered ineffective against Gram-negative bacteria. This is generally attributed to the presence of the outer membrane or peptidoglycan layer which hinders access to the plasma membrane (Nybroe and Sørensen, 2004; Raaijmakers et al., 2006). However, things are not as clear-cut since CLPs of the tolaasin group (Lo Cantore et al., 2006) and the recently described xantholysin group (Li et al., 2013; Molina-Santiago et al., 2015) are able to inhibit Gram-negative bacteria. Furthermore, WLIP-(V) showed a modest activity against the Gram-negative *Erwinia carotovora* subsp. *carotovora* (Lo Cantore et al., 2006). To add to the apparent confusion, conflicting activities have been reported against some *Xanthomonas* species, whereby WLIP-(V) did (Rokni-Zadeh et al., 2012) or did not (Lo Cantore et al., 2006; Andolfi et al., 2008) show activity against this Gram-negative bacterium. This notwithstanding, it is clear that the activity of Ps-CLPs should not be considered as limited to Gram-positive bacteria.

### Bacterial Mobility

In addition to the antagonistic effects against bacteria that were described above, Ps-CLPs are also involved in controlling the motion of their producers. Mobility can occur by a range of mechanisms including swarming, swimming, twitching, gliding and sliding (Kearns, 2010). Swarming often depends on the reduction of the critical surface tension of liquids (Xu et al., 2012). In this respect, secretion of biosurfactants such as CLPs potentially reduces the drag of bacterial cells during swarming, facilitating lateral movement across the surface (Deziel, 2003). Most of the CLPs produced by *Pseudomonas* spp. are able to reduce the surface tension of growth media to different extents (Fechtner et al., 2011). A minimal threshold surface tension of 24.16 mN m^-1^ was calculated for *Pseudomonas* bacteria, based on a diverse set of surface tension reducing isolates (Fechtner et al., 2011; Mohammed et al., 2015). This threshold is likely set by limits in, among others, biosynthesis and transport of surfactants out of the bacterial cell. For comparison, Triton X100, often used as laboratory detergent reduces the surface tension to 30 mN m^-1^. Arthrofactin-(A) appears to be the most powerful biosurfactant, lowering the surface tension to 24.1 mN m^-1^ at a CMC of 7.4 μmol L^-1^ (Morikawa et al., 1993). Ps-CLPs with much longer peptide chains have a much higher CMC and cause a smaller reduction in surface tension. For example, tolaasin and syringopeptin 22A reduce the surface tension to 38 mN m^-1^ and 40 mN m^-1^ at CMCs of 211 and 433 μmol L^-1^, respectively (Hutchinson and Johnstone, 1993; Hutchinson et al., 1995; Roongsawang et al., 2010).

Several studies have focussed on Ps-CLPs and their involvement in surface motility of the producing *Pseudomonas* strains either on semi-solid agar (Andersen et al., 2003; Roongsawang et al., 2003; De Bruijn et al., 2007, 2008; D’Aes et al., 2010, 2014) or in their natural habitats (Hildebrand et al., 1998; Nielsen et al., 2005; Tran et al., 2007; Alsohim et al., 2014). By using CLP-deficient mutants, their role in the motility of *Pseudomonas* species was analyzed. In most cases, surface motility on semi-solid agar plates was lost for the CLP-deficient mutants, while parent strains did show motility (**Table 1**). Generally, motility is restored when endogenous lipopeptides are added externally to the growth medium used for Ps-CLP deficient species, conclusively showing that lipopeptide-production is responsible for bacterial motility. Interestingly, for an amphisin-deficient mutant of *Pseudomonas* sp. DSS73, addition of structurally related [amphisin, tensin-(A), viscosinamide-(V)] biosurfactants from other *Pseudomonas* strains also restored bacterial motility while biosurfactants from unrelated species, for instance serrawettin from *Serratia marcescens*, did not (Andersen et al., 2003). Also, synthetic surfactants (NP40 and Triton X-100) failed to restore the motility on semi-solid agar plates. Together this indicates that reduction in surface tension by itself is insufficient and that other physical-chemical properties of the biosurfactants are crucial for surface motility.

**Table 1:** Overview of swarming properties of lipodepsipeptide-producing *Pseudomonas* bacteria and their deficient mutants.

Bacterial species	Lipodepsipeptide	Swarming	Motility in CLP-deficient mutant	Motility upon addition CLP	Reference
*P.* sp. SBW25	Viscosin	Yes	Lost	Restored	De Bruijn et al., 2007; Alsohim et al., 2014
*P.* sp. DR54	Viscosinamide-(V)	Yes	N.D.	N.D.	Nielsen et al., 2002
*P*. *putida* RW10S2	WLIP-(V)	Yes	Lost	N.D.	Rokni-Zadeh et al., 2012
*P.* sp. SS101	Massetolide-(V)	Yes	Lost	N.D.	De Bruijn et al., 2008
*P. fluorescens* Pf-5	Orfamide A	Yes	Lost	Restored	Gross et al., 2007; D’Aes et al., 2014; Ma et al., 2016a
*P. fluorescens*	Orfamide A	yes	Lost	Restored	Flury et al., 2017
*P. poae* RE^∗^1-1-14	Poaeamide-(O)	Yes	Lost	N.D.	Zachow et al., 2015
*P.* sp. DSS73	Amphisin	Yes	Lost	Restored	Koch et al., 2002; Andersen et al., 2003
*P. fluorescens* HKI0770	Anikasin-(A)	Yes	Lost	N.D.	Klapper et al., 2016; Gotze et al., 2017
*P.* sp. MIS38	Artrofactin-(A)	Yes	Lost	Reduced	Roongsawang et al., 2003
*P.* sp. PCL1445	Putisolvin	Yes	Reduced	N.D.	Kuiper et al., 2003
*P. syringae* pv. *tomato* DC3000	Syringafactin	Yes	Lost	Restored	Berti et al., 2007; Burch et al., 2012
*P. entomophila*	Entolysin	Yes	Lost	N.D.	Vallet-Gely et al., 2010
*P. putida* BW11M1	Xantholysin	Yes	Lost	N.D.	Li et al., 2013
*P. tolaasii*	Tolaasin	No	No	N.D.	Henkels et al., 2014
*P.* sp. CMR12c	Sessilin-(T)	No	No	N.D.	D’Aes et al., 2010


**FIGURE 3:**
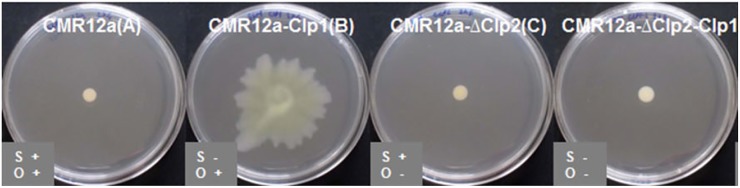
Bacterial swarming of *Pseudomonas* sp. CMR12a on 0.6% SSM agar plates, and that of mutants deficient in sessilin-(T) (CLP1), orfamide (CLP2), or sessilin+orfamide (CLP1+CLP2) production. Figure reprinted from D’Aes et al. (2014) with permission.

When the *Pseudomonas tolaasii* (producing tolaasin) is exposed to *P. ‘reactans’* LMG 5329 on agar, the formation of a white line precipitate is observed, a feature that was subsequently attributed to the White Line Inducing Principle (WLIP), a Ps-CLP from the viscosin group produced by *P. ‘reactans’* LMG 5329. Interaction is accompanied by a reduced surface motility of its parent *Pseudomonas* strain. While originally attributed to WLIP-(V) only, a white line reaction involving tolaasins [or sessilins-(T)] can also occur with orfamides (D’Aes et al., 2014; Henkels et al., 2014). CLPs which are more closely related to WLIP such as viscosin and massetolide-(V) do not appear to trigger such a reaction, indicating a structure-dependent interaction between the two CLPs (Rokni-Zadeh et al., 2013). It was described that *Pseudomonas* sp. CMR12a, which produces both sessilins-(T) and orfamides, is static while the sessilin-deficient mutant is able to swarm (**Figure 3**). This swarming, most likely enabled by orfamide production, is inhibited by the addition of sessilin producers. However, the interaction dynamics has additional complexity since adding sessilin containing supernatant did not block the motility (D’Aes et al., 2014; Olorunleke et al., 2017).

### Fungi and Oomycetes

A large body of research has focussed on the antifungal and anti-oomycete properties of CLPs. Orfamide A, nunapeptin-(SP) or sessilin-(T) had little or no detectable effect on the growth or morphology of hyphae of *Rhizoctonia solani* while for viscosinamide-(V), pseudophomin-(V), tensin-(A), pseudomycin-(SM), the tolaasins, entolysin, and nunamycin-(SM) growth inhibition was clearly observed (Harrison et al., 1991; Hansen et al., 2000; Nielsen et al., 2000; Pedras et al., 2003; Bassarello et al., 2004; Gross et al., 2007; Kruijt et al., 2009; Michelsen et al., 2015b; Ma et al., 2016a). Remarkably, co-production of orfamide A and sessilin-(T) by *Pseudomonas* sp. CMR12a did inhibit growth of *R. solani* whereas individually, they did not (Olorunleke et al., 2015a). The pseudophomins-(V), the pseudomycins-(SM), and WLIP-(V) all displayed antifungal activity against the phytopathogens *Sclerotinia sclerotiorum* and *Phoma lingam* (Pedras et al., 2003; Lo Cantore et al., 2006). Syringomycin E inhibited growth of the postharvest green mold of citrus fruits (*Penicillium digitatum*) (Bull et al., 1998). In addition, this lipopeptide showed antagonistic activities against the human-pathogenic fungi *Aspergillus flavus, A. niger, A. fumigatus, Fusarium moniliforme*, and *F. oxysporum* (De Lucca et al., 1999). Finally, WLIP-(V), putisolvin, syringotoxin-(SM), syringopeptin 22A, syringopeptin 25A, and syringomycin E and tolaasin all inhibit the fungal pathogen *Botrytis cinerea* with different potencies (Grgurina et al., 2005; Andolfi et al., 2008; Kruijt et al., 2009).

**FIGURE 4:**
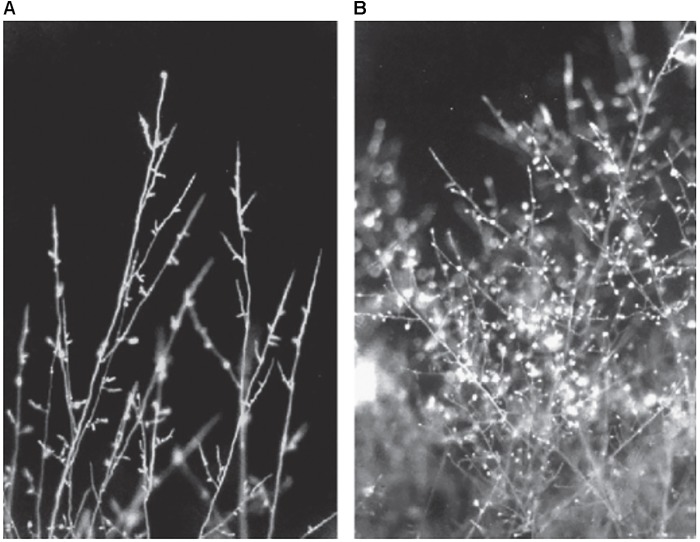
Cell walls and septa of *R. solani* stained with Calcofluor white. **(A)** Untreated fungus shows straight hyphae with only few branches. **(B)** Treatment with viscosinamide lead to appearance of highly branched hyphae. Figure reprinted from Raaijmakers et al. (2010) with permission.

In the presence of purified viscosinamide-(V), *R. solani* showed stunted growth, increased branching and hyphal swelling (**Figure 4**; Raaijmakers et al., 2010). Viscosinamide A-(V) has a similar effect on *Pythium ultimum.* Also, *Pseudomonas* species producing viscosin, poaeamide-(O), amphisin, lokisin-(A), tensin-(A), syringomycin E, and sessilin-(T) were reported to inhibit mycelial growth of *P. ultimum* or *Phytophthora infestans* oomycetes *in vitro* (Nielsen et al., 2000, 2002; Naseby et al., 2001; Andersen et al., 2003; De Bruijn et al., 2007; Van De Mortel et al., 2009; D’Aes et al., 2014; Zachow et al., 2015; Kawasaki et al., 2016) while production of entolysin is not correlated with biocontrol activity of *P. ultimum* (Vallet-Gely et al., 2010). Massetolide A-(V) was found to inhibit mycelial growth of *P. infestans* but not of *P. ultimum* (Mazzola et al., 2007; De Bruijn et al., 2008).

Several experiments addressing protection from fungi *in planta* with *Pseudomonas* spp. have been described in literature as well. The viscosinamide-producing *P. fluorescens* DR54 caused an increased plant emergence of sugar beet from *P. ultimum*-challenged seeds, although the levels of the control experiments were not reached (Nielsen et al., 1998). No increase in root length was observed for non-challenged seeds in the presence of *P. fluorescens* DR54. Similarly, massetolide-producing *P. fluorescens* SS101 prevents and reduces infection of tomato leaves by *P. infestans*. A CLP-deficient mutant was significantly less effective in biocontrol, while purified massetolide A-(V) displayed significant control of the pathogen (Tran et al., 2007). Similar results were obtained with *P. fluorescens* SBW25 and its viscosin-deficient mutants.

The yeast-like fungi *Cryptococcus neoformans* and *Candida albicans* were found to be inhibited by tolaasin (Andolfi et al., 2008), anikasin-(A) (Gotze et al., 2017), and the pseudomycins-(SM) (Harrison et al., 1991) but not by viscosin (Geudens et al., 2017) or some of its group members including WLIP (Lo Cantore et al., 2006; Andolfi et al., 2008), pseudodesmin A (Sinnaeve et al., 2009b), viscosinamide (Geudens et al., 2017) or by pseudofactin II (Janek et al., 2012) or syringopeptin 22A (Grgurina et al., 2005). Finally, thanamycin-(SM), featuring the exceptionally rare occurrence of hydroxylation at the Orn2 C^α^, is 32 times more potent compared to the related syringomycin E (Johnston et al., 2015).

Although interesting antifungal properties have mostly emerged from work relating to crop protection and biocontrol, they could also present an important therapeutic relevance as antifungal drugs. Unfortunately, purified Ps-CLPs can be haemolytic, such as reported for massetolide A-(V) (Van De Mortel et al., 2009), WLIP-(V) (Andolfi et al., 2008), orfamide A (Henkels et al., 2014), arthrofactin (Morikawa et al., 1993), tolaasins (Rainey et al., 1991), syringomycin E (Agner et al., 2000), and syringopeptins 22A-B (Hutchinson and Gross, 1997). Despite this, a single injection of 10 mg kg^-1^ of massetolide A was reported to be non-toxic to mice (Gerard et al., 1997). Pseudomycin B, a CLP of the syringomycin group, is effective against pathogenic fungi in mice and rats, but causes irritation and eventually necrosis of the affected tissues (Sun et al., 2001). A series of pseudomycin analogs or prodrugs were obtained by derivatization of the natural compound. These compounds retained antifungal effects *in vitro* and *in vivo*, but show less toxicity to experimental animals (Chen et al., 2000; Jamison et al., 2000; Sun et al., 2001; Zhang et al., 2001a,b). These analogs and prodrugs are now patented by Eli Lilly and Company (Chen et al., 2001; Kulanthaivel et al., 2003).

### Protozoa

Several secondary metabolites, for example lipodepsipeptides produced by rhizobacteria, are able to provide protection against grazing by different protozoan species. The presence of certain Ps-CLPs can lead to lysis of various protozoa, as shown for viscosin (Burke et al., 1999), viscosinamide A (Andersen and Winding, 2004; Pedersen et al., 2011), amphisin (Pedersen et al., 2011), anikasin-(A) (Gotze et al., 2017), and syringomycins (Takemoto et al., 2001).

The ability to lyse protozoan predators represents an important natural function for lipopeptides: soil populations of CLP-deficient *Pseudomonas* strains are compromised in the presence of protozoa compared to that of soils with the CLP-producing parental strains (Mazzola et al., 2009). Moreover, exposure of massetolide-(V) producing *P. fluorescens* SS101 or viscosin-producing *P. fluorescens* SBW25 to *N. americana* resulted in twofold upregulation of genes involved in CLP biosynthesis (Mazzola et al., 2009; Song et al., 2015). This suggests that bacteria can modulate the production of secondary metabolites in response to protozoan predators. Possibly, this constitutes a defense mechanism for bacterial strains when confronted with protozoa.

### Insects

Cyclic lipodepsipeptide-producing *Pseudomonas* spp. are mostly analyzed for their ability to inhibit bacterial, fungal or protozoan growth. However, insecticidal properties have also been observed. Strong insecticidal activity seems to be correlated with the production of the *P. fluorescens* insecticidal toxin (Fit), a sensor protein responsible for the detection of the host environment (Pechy-Tarr et al., 2013; Ruffner et al., 2013). It activates insecticidal toxin production specifically upon infection of the insect host but not while on plant roots or in standard laboratory media (Pechy-Tarr et al., 2013; Kupferschmied et al., 2014). However, the toxin is not the unique killing factor as mutants lacking the Fit genes still caused a substantial mortality (Ruffner et al., 2013; Rangel et al., 2016). The insecticidal activity of *Pseudomonas* species appears highly multifactorial whereby multiple antimicrobial compounds, including CLPs, contribute to the oral insecticidal activity (Keel, 2016; Loper et al., 2016; Flury et al., 2017; Lim et al., 2017). Using mutant bacterial strains deficient in CLP production, it was determined that orfamide A and B, and sessilin-(T) all contribute to the (oral) insect toxicity exhibited by their producer strains. When applied topically, a dose-dependent mortality was also observed for purified orfamide A against aphids (*Myzus persicae*) (Jang et al., 2013). Additionally, insecticidal activity against aphids has also been reported for viscosin (Kupferschmied et al., 2013). Remarkably, whereas the entolysin-producing *P. entomophila* is able to infect and kill *Drosophila melanogaster* upon ingestion, entolysin itself was reported not to participate in its virulence (Vallet-Gely et al., 2010).

### Viruses

There is very little information available about the possible role in nature of the antiviral activity of CLPs, mostly because there is no clear link between the natural habitat of CLP-producing bacteria and the presence of viruses. As a result, data on antiviral activity of Ps-CLPs appears quite sparse. Nevertheless, the very first report on the lipodepsipeptide viscosin in 1951 already mentioned the inhibition and inactivation of human pathogenic viruses such as the infectious bronchitis virus, influenza A virus, and Newcastle disease virus (Groupé et al., 1951). Recently, Shimura et al. (2013) described the antiviral activity of the xantholysin-like lipopeptide MA026 against Hepatitis C.

### Impact of Ps-CLPs on Plants

*Pseudomonas* are often found colonizing the rhizosphere, where they can either be pathogenic or beneficial for plant growth. Thus, it is not surprising that many investigations have focussed on their plant potential for biocontrol or biostimulation. Considerable data exists that indicates a direct or indirect role of Ps-CLPs in this respect. Experiments performed *in planta* for *P. fluorescence* SBW25 and its viscosin-deficient mutant showed that sugar beet or pea seedlings did not display different germination or development when pre-soaked in water, wild-type or mutant bacterial solutions, demonstrating that viscosin alone does not promote plant growth (Naseby et al., 2001; Alsohim et al., 2014). Similar observation have been made for massetolide A-(V) (Tran et al., 2007; Cheng et al., 2017). However, some CLPs are involved in the virulence of certain plant pathogens, although it is believed that they only contribute to the colonization of the plants, rather than participate in the virulence itself. For example, viscosin production is required for the plant-pathogenic *P. fluorescens* 5064 to spread to adjacent non-wounded broccoli florets (Hildebrand et al., 1998). Additionally, while plant infection of *P. syringae* pv. *syringae* still occurs in the absence of syringomycin or syringopeptin, disease severity increases drastically when these CLPs are also present (Scholtz-Schroeder et al., 2001).

**FIGURE 5:**
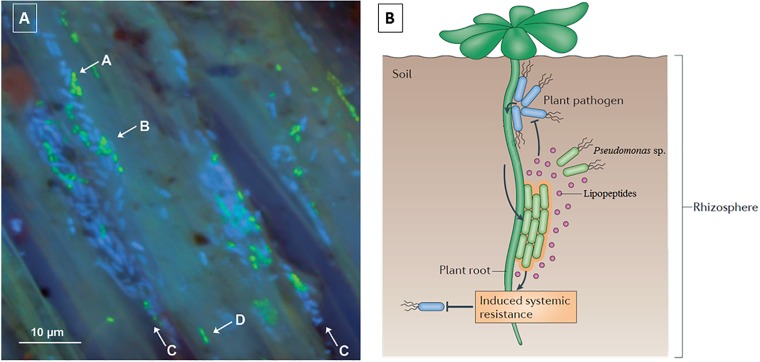
**(A)** Localization of gfp-*Pseudomonas* sp. DR54 (green cells) and indigenous bacteria (blue cells) in the rhizosphere (rhizoplane) of barley roots. Mixed micro-colonies are indicated by arrows A and B. Epithelial cells are indicated with arrows C and D. Figure modified from Normander et al. (1999); **(B)** Schematic of the biocontrol mechanisms of CLPs, including induced systemic resistance. Figure reprinted from Vlamakis et al. (2013) with permission.

Plant biocontrol relies partly on the production of lipodepsipeptides since these molecules exert their antagonistic effects against soil borne pathogens. Many different *Pseudomonas* spp. can be isolated from the rhizosphere of plants, where they are able to form biofilms on the root surfaces. For example, green fluorescent protein (*gfp*) labeled *Pseudomonas* sp. DR54, which produces viscosinamide A-(V), is found in the rhizosphere (rhizoplane) of barley roots (**Figure 5A**), forming mixed micro-colonies together with the indigenous bacteria located near the crevices of epithelial cells of the roots (Normander et al., 1999). Additionally, there is evidence that CLPs are able to induce systemic resistance (ISR) in plants, providing an additional defensive effect against physically separated pathogens (**Figure 5B**). ISR involves the stimulation of the immune systems in plants and is an alternate mechanism by which *Pseudomonas* spp. can induce biocontrol against fungi and oomycetes (Tran et al., 2007; Ma et al., 2016b).

### Biofilms

Cyclic lipodepsipeptides have also been implied in the regulation of biofilms and thus hold potential for biomedical applications, for instance as part of coatings that prevent biofilm formation. Thus, considerable literature relating to the effect of Ps-CLPs on biofilms is also available. For *Bacillus* and *Pseudomonas* spp., CLPs play an important role in surface attachment and biofilm formation, although the outcome depends on the type of CLP (Raaijmakers et al., 2010). Massetolide A-(V) (De Bruijn et al., 2008), sessilin-(T) (D’Aes et al., 2014), and xantholysin (Li et al., 2013) support their producers in the formation of biofilms, whereas viscosin (Bonnichsen et al., 2015), WLIP (Rokni-Zadeh et al., 2012), orfamide B (D’Aes et al., 2014), arthrofactin (Roongsawang et al., 2003), and putisolvin I/II (Kuiper et al., 2003; Kruijt et al., 2009) appear to inhibit biofilm formation of their producing strains. Additionally, entolysin (Vallet-Gely et al., 2010) does not appear to be involved in the formation or breakdown of biofilms, although it should be mentioned that no detailed time-resolved experiment was performed, possibly explaining this apparent difference.

Initially, a single time-point analysis showed that viscosin is involved in the formation of biofilms by *Pseudomonas* sp. SBW25, since its viscosin-deficient mutants were unable to form biofilms on an artificial surface. Supplementing the mutant’s growth medium with purified viscosin restored biofilm formation (De Bruijn et al., 2007). However, in a subsequent time-resolved study (Bonnichsen et al., 2015) it was shown that biofilms of a viscosin-deficient mutant and its wild-type *P.* sp. SBW25 developed comparably during the first 11.5 h. Subsequently, a significant difference emerged as the amount of *P.* SBW25 associated biofilm decreased, whereas the mutant continued to develop biofilm throughout the period. Thus, over time, the viscosin-deficient mutant forms *more* biofilm than the wild-type in long-term incubations, in contrast with what was found earlier (De Bruijn et al., 2007). The authors attributed this to the temporal dynamics of biofilm formation and dispersal. When dispersal of biofilms was induced through carbon starvation, a viscosin-deficient mutant dispersed to a lesser extent than the wild-type. Notably, carbon starvation did partially induce viscosin biosynthesis gene expression in the wild-type, followed by biofilm dispersal. The dispersal mechanism is of vital importance as it allows bacteria to escape from unfavorable nutrient conditions and spread throughout the environment to colonize new areas.

Given the variable and sometimes contradictory effects in biofilm regulation noted to date from only a handful of Ps-CLPs, the underlying mechanisms in biofilm formation and development can only be speculated upon. It is possibly related to the differing physicochemical properties and to the potential effect of CLPs on the cell surface and/or substrate (Neu, 1996; Raaijmakers et al., 2010). Indeed, hydrophobic interactions and surface-active compounds, including CLPs, have been suggested to play a role in the adherence of cells to surfaces (De Bruijn et al., 2008). For instance, whereas pseudofactin II did not present any antifungal activity against *C. albicans*, it did show a marked ability to reduce adhesion and biofilm formation of this pathogen on catheters or polystyrene surfaces pre-incubated with solutions of pseudofactin II above its critical micelle concentration. Similar results were obtained for *Staphylococcus aureus, S. epidermidis* and *Streptococcus agalactiae*. In addition, pseudofactin II is able to disperse preformed biofilms of all these pathogens (Biniarz et al., 2015; Janek et al., 2016).

### Anti-proliferative Activities of Ps-CLPs

Another domain which is opening up to Ps-CLPs is that of cancer research. For example, it was observed that viscosin inhibits migrations of a breast cancer cell line (MDA-MD-231) and a metastatic prostate cancer cell line (PC-3M) without causing toxicity (Saini et al., 2008). Pseudofactin II was reported to induce apoptosis of human melanoma cells while normal human dermal fibroblast cells, used as a control, were less affected (Janek et al., 2013). The authors hypothesized that apoptosis might be induced by interaction of lipopeptide micelles with the plasma membrane, leading to membrane permeabilization.

When various cancer cell lines (T-cells leukemia, mantle cell lymphoma, and melanoma cell lines) were treated with a mixture of the syringomycin-group CLP nunamycin and nunapeptin, a syringopeptin group member, apoptosis ensued while treatment of healthy cells in the same concentration range had no effect. After treatment with the CLP mixture, the majority of the leukemia T-cells seemed to be in the early apoptotic state. Treatment with either Ps-CLP alone did not prompt apoptosis (Michelsen et al., 2015a).

The major lipopeptide isolated from *Pseudomonas soli* sp. nov., identified as xantholysin A, and MDN-0066, isolated from *Pseudomonas granadensis* F-278,770^T^ showed activity on a kidney tumor cell line (RCC4) (Pascual et al., 2014; Cautain et al., 2015). The authors attributed this selective toxicity to an inactivation of the Von Hippel Lindau (VHL) gene linked to activation of the hypoxia induced transcription factor (HIF) pathway.

### Mechanism of Action of Ps-CLPs

Considerable literature supports or indicates membrane-perturbation – and in particular pore-formation – as the origin of antimicrobial activity. This is in line with the amphipathic character of Ps-CLPs, which enables these peptides to perturb the barrier function of the membrane. Indeed, several Ps-CLPs are able to permeabilize model membranes with varying composition, possibly though formation of transmembrane pores (Brodey et al., 1991; Cho and Kim, 2003; Lo Cantore et al., 2006; Andolfi et al., 2008; Geudens et al.). These pores are thought to collapse the pH gradient across the membrane by increasing the influx of H^+^ and Ca^2+^ ions as well as the efflux of K^+^ ions (Kozlova et al., 2004; Aiyar et al., 2017). This ultimately leads to an induction of calcium-mediated signaling pathways, leading to cell death. However, the precise events involved in the pore-forming processes of Ps-CLPs and the exact type of pores remains largely unknown. A variety of membrane perturbing mechanisms for membrane-active, often cationic antimicrobial peptides, have been described and the reader is referred to excellent reviews on this topic (Brogden, 2005; Melo et al., 2009; Lee et al., 2016; Li et al., 2017; Kumar et al., 2018). However, the overall bulk of studies relates to cationic antimicrobial peptides, and few systematic studies are available for Ps-CLPs. For syringomycin E, a body of published research indicates a role for fungal sphingolipids in its antifungal action (Bender et al., 1999; Stock et al., 2000; Takemoto et al., 2003; Kaulin et al., 2005). More specifically, the presence of sphingolipids with natural (C4-hydroxylated) long chain bases are a requisite for the yeast *Saccharomyces cerevisiae* to be sensitive *in vivo* to syringomycin E (Stock et al., 2000; Kaulin et al., 2005). A yeast mutant having sphingolipids with a sphingoid base devoid of C4-hydroxylation was found to be resistant to the lipopeptide. Interestingly, the presence of the mutant sphingolipid diminished the ability of syringomycin E pores to open synchronously but it did not alter the single pore conductance or the gating charge (Kaulin et al., 1998). In another notable study, five Ps-CLPs from different groups were investigated for their interactions with bacteria-like lipid model membranes Reder-Christ et al. (2012). It was proposed that their membrane incorporation is driven by the initial interaction of the polar amino acid residues with the polar head groups of the phospholipids, followed by incorporation of the fatty acids into the membrane core. Subsequently, the lipopeptide is assumed to incorporate into the membrane. This incorporation is reversible, although the dissociation rate constant is low compared to the association rate (Reder-Christ et al., 2012). In other work, total synthesis of the enantiomer of pseudodesmin A, a member of the viscosin group, was used to exclude chiral, receptor-based interactions as the main determinant of the biological activity since the enantiomer proved as active as the natural compound (De Vleeschouwer et al., 2014). Despite these reports, the detailed molecular events leading to pore-formation, once individual molecules have been incorporated, and the exact relation to Ps-CLP sequence and structure remains to be established. In this respect it was proposed that the self-assembly of viscosin group Ps-CLPs potentially plays an (indirect) role in the biological activity of these CLPs, as it could be an import factor in the creation of pore-like structures through the cellular membrane (Sinnaeve et al., 2009a; Geudens et al., 2014). Also, Syringomycins actively involve lipid molecules in the structural assembly process leading to pore-formation (Malev et al., 2002; Szabó et al., 2004). It is believed that an asymmetric lipid pore is formed that is stabilized by peptide molecules (Malev et al., 2002; Ostroumova et al., 2007a). Therefore, the presence of different lipids and/or sterols can modulate the effect of the CLPs (Takemoto et al., 1991; Julmanop et al., 1993; Wangspa and Takemoto, 1998; Dalla Serra et al., 1999; Coraiola et al., 2006; Efimova et al., 2016). Additionally, the membrane dipole potential appears to play a significant role in the pore forming ability of Ps-CLPs (Ostroumova et al., 2007b, 2008). Nevertheless, when considering the sequence and structure of the various Ps-CLP groups in **Figure 1**, it is unlikely that a single overall mode of action can be attributed to explain their bioactivities. For instance, syringomycins are multiply positively charged lipopeptides, with a predominance of polar, hydrophilic residues, whereas most others are neutral lipopeptides or feature a single negative charge while apolar, hydrophobic residues are prevalent (**Figure 1**). Starting with tolaasin, the larger Ps-CLPs display a single positive charge occurs within the macrocycle, with either a long amphipathic oligopeptide sequence in the case of tolaasin or a mostly hydrophobic one for the other Ps-CLP groups characterized by long exocyclic oligopeptide moieties. Thus, while considerable data is now available, much terrain remains to be covered. It is hoped that the growing interest in Ps-CLPs, combined with the biological data reviewed here and the further development of efficient synthesis route to Ps-CLP will stimulate detailed biophysical studies into their mechanism of action.

## Discussion

**FIGURE 6:**
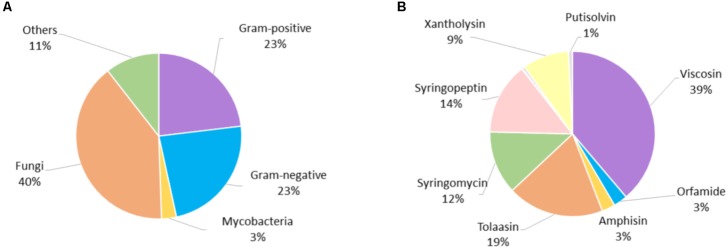
**(A)** Distribution of biological tests according to micro-organism type, **(B)** distribution of all biological tests according to CLP group.

We attempted to exploit the material collected for this review to gain additional insight into general trends and correlations in the biological activity of Ps-CLPs (**Supplementary Table 1**). As most data pertains to antibacterial and antifungal properties, only these were analyzed in more detail. In total, 66 literature reports (**Supplementary Figure 1**) were used to analyze a total of 775 biological tests, involving 51 CLPs, of which 507 (65%) showed a clear activity compared to controls. Significantly more tests were performed against fungi (306; 39%) compared to Gram-positive (180; 23%) and Gram-negative bacteria (181; 23 %), whereas more limited testing was described with respect to mycobacteria (22; 3%) (**Figure 6A**).

Although Ps-CLPs have been described since the 1950s, it is only since the mid-1990s that routine testing of the biological functions of Ps-CLPs started (**Supplementary Figure 2**). The use of a particular category of micro-organisms for tests involving Ps-CLPs does not show any specific trend with time (data not shown).

Of the 14 Ps-CLP groups that were considered (**Figure 1**), the viscosin group is the most studied, being the subject of 39% of all biological assays (**Figure 6B**). However, this is mainly due to the high number of tests performed for WLIP: 160 of the 298 tests for the viscosin group involve this particular Ps-CLP and overall it represents just over one fifth (20%) of the assays involving all Ps-CLP reviewed here. Thus, it is the best characterized Ps-CLP (*vide infra*). The surprisingly large number of tests can indicate some bias in the environments sampled but also reflects the interest in the “White Line” reaction it forms with the tolaasins (50% of WLIP tests). Indeed, the latter Ps-CLP group has also been extensively tested (145 tests), representing 19% of all assays involving Ps-CLP. At the other extreme, four Ps-CLP groups (the orfamides, amphisins, putisolvins and pseudofactins) each represent less than 2.5% of all assays. Thus, there is quite some variation in the extent to which specific the activities of individual Ps-CLPs have been uncovered. Within each category of micro-organisms, the distribution of tests per CLP group is similar to that shown in **Figure 6B**, although slightly more tests against Gram-positive bacteria have been performed for viscosin group Ps-CLPs (**Supplementary Figure 3**).

Despite the high number of available biological tests, it is difficult to use these for a comparative overview, let alone to correlate the absolute and relative activities of CLPs with certain structural features. Difficulties arise mainly from the lack of overlap in the micro-organisms used for testing and from differences in assay types and resulting quantification of bioactivity using different measures (inhibition zones, inhibitory concentrations, inhibitory quantities etc.). As part of their investigation into antibiotic activities of *Pseudomonas* lipopeptides using model membrane systems, Reder-Christ et al. (2012) investigated the antimicrobial activity of massetolide A-(V), syringomycin E, orfamide A, arthrofactin-(A), and entolysin B against 16 Gram-positive species over 8 genera and 6 Gram-negative species over 5 genera using a disk diffusion assay. Of these 110 tests, only 8 proved positive, all against Gram-positive bacteria, with all CLPs but orfamide being active against *Arthrobacter crystallopoietes* DSM 20117, and only arthrofactin-(A) showing additional activity against *Corynebacterium* (3 species) and *Mycobacterium smegmatis* ATCC 70084. None of the Ps-CLPs proved active against the Gram-negative bacteria. The lack of activity appears at odds with the other literature data reported here. Closer inspection with other reports of diffusion disk assays shows that the quantities used for testing (1.5 to 3 μg) are to the low end compared to those used in other work, which typically ranges from 9 to 50 μg (Grgurina et al., 2005; Li et al., 2013). The concentration used for such assays, is obviously set by what is considered to be a useful threshold to indicate weak, medium or strong antimicrobial activity. As we used a binary grading (*vide infra*) to survey activities of Ps-CLPs and the test reported by Reder-Christ et al. (2012) uses low test concentrations to detect significant biological activity, we chose not to include their data in the number presented in our survey.

**FIGURE 7:**
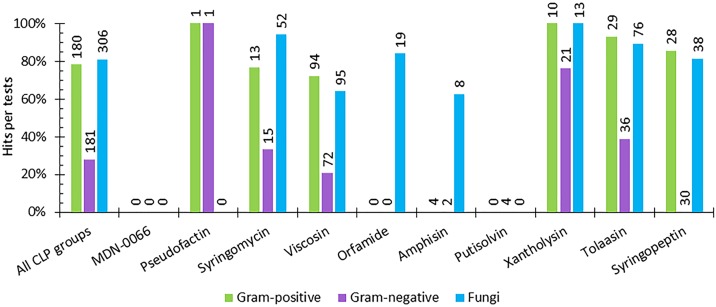
The percentage of hits per activity test against Gram-positive, and Gram-negative bacteria and against fungi for each Ps-CLP group investigated so far. The numerical data labels above the individual series indicate the total number of tests performed. If all tests are hits, 100% is reached.

In the following, we consider the biological activities of Ps-CLPs against Gram-positive, Gram-negative and fungi in a binary fashion, where a Ps-CLP is simply active (“Hit”) or inactive (“Miss”) compared to the control, irrespective of the assay used (**Figure 7** and **Supplementary Figure 4**).

Not unexpectedly, the initial survey of the data in **Figure 7** suggests that mapping of the biological activity spectrum is still quite fragmented, both in scope and in number. Understandably, the more limited activity profiling simply reflects the specific interest of the authors. Nevertheless, some patterns in activity profiles can be seen.

For the 7 Ps-CLP groups that were tested for their antifungal properties, activity against 41 different fungal genera (representing 72 different species) is apparent in 81% of the tests, indicating a clear antifungal potential throughout the Ps-CLPs (**Figure 7** and **Supplementary Table 2**). Considering only those micro-organisms for which the number of tests *n* per CLP group equals 3 or more (*n* ≥ 3) on a genus-level (e.g., *Aspergillus* or *Fusarium*), the Ps-CLPs generally display a broad activity against fungi (**Table 2**). In total, 95 biological tests were used to assess the antifungal properties of viscosin group Ps-CLPs, of which 65% were positive. Since several genera did not satisfy our *n* ≥ 3 test criterion (vide supra), they are not included in our analysis. However, more extensive tables are available in the **Supplementary Information**. Ten different fungal genera were challenged with viscosin group Ps-CLPs with *n* ≥ 3, of which only three were unaffected: *Aspergillus* (*n* = 4), *Candida* (*n* = 10), and *Cryptococcus* (*n* = 4). The viscosin group CLPs showed clear activity against *Phytophthora infestans* (*n* = 4), but not against *P. citrophthora* (*n* = 2) or *P. nicotianae* (*n* = 2). Another set of ten fungal genera, partly overlapping with those challenged with viscosin, were exposed to syringomycin group members, showing activity against all. No conclusions can currently be drawn regarding the activity of Ps-CLPs from other groups against *Aspergillus* or *Cryptococcus*, as these were not tested (or *n* < 3). Ps-CLPs of the syringopeptin groups also display activity against *Candida* (*n* = 8), while some selectivity seems present for tolaasins (*n* = 4). Of the 9 fungal genera treated with tolaasins, only *Candida* showed some resistance. More specifically, a clear activity was reported against *C. albicans* (*n* = 2), but *C. parapsilosis* was unaffected (*n* = 2).

**Table 2:** Overview of the number of “hits” compared to the number of tests *n* reported against fungi per Ps-CLP group.

	Syringomycin	Viscosin	Orfamide	Xantholysin		Tolaasin	Syringopeptin
*Agaricus*						4 (*n* = 4)	
*Aspergillus*	7 (*n* = 10)	0 (*n* = 4)					
*Botrytis*	3 (*n* = 3)						
*Candida*	7 (*n* = 7)	0 (*n* = 10)				2 (*n* = 4)	8 (*n* = 8)
*Cryptococcus*	7 (*n* = 7)	0 (*n* = 4)					
*Fusarium*		4 (*n* = 6)		5 (*n* = 5)		6 (*n* = 6)	
*Geotrichum*	6 (*n* = 6)						3 (*n* = 6)
*Lentinus*						3 (*n* = 3)	
*Phoma*		3 (*n* = 3)					
*Phytophthora*	4 (*n* = 4)	4 (*n* = 8)	4 (*n* = 4)				
*Pleurotus*	9 (*n* = 9)	4 (*n* = 5)					
*Pythium*		14 (*n* = 14)	3 (*n* = 4)				
*Rhizoctonia*	9 (*n* = 9)	8 (*n* = 8)	5 (*n* = 6)				
*Rhodotorula*	7 (*n* = 10)			19 (*n* = 23)			6 (*n* = 6)
*Sclerotinia*	4 (*n* = 4)	6 (*n* = 6)					
*Magnaporthe*			3 (*n* = 3)				


Five fungal genera were tested against at least three Ps-CLP groups with excellent antifungal activity, with the exception of the viscosin group against *Candida*. All other CLP groups, tested with 2 to 4 fungal genera also showed activity against all tested fungi, indicating a broad spectrum effect. In addition, the susceptibility of Gram-positive bacteria against Ps-CLPs already evident from individual studies appears as an almost generic trait, based on the data collected here.

More specifically, 180 tests were performed to assess the activity of Ps-CLPs against 18 different genera (representing 34 species) of which 141 tests were positive (78%) (**Figure 7**). The collected data allows to identify 6 Ps-CLP groups where the number of tests *n* ≥ 3 on a genus-level involving 11 genera (**Table 3**). The data shows an almost generic impact on Gram-positive bacteria of the 5 Ps-CLP groups. *Bacillus* spp. are found to be strongly affected (88% of the tests on average) by all Ps-CLPs, while all 9 genera listed are strongly affected by viscosin group Ps-CLPs. The only exception is *Staphylococcus* where hits were observed for ∼50% of all tests. This is also the case for *Micrococcus*, although this should be confirmed as *n* = 4 only. While for the other 4 Ps-CLP groups biological activities are reported in a much more focused set of 2 or 3 genera, activity is present at 90% or more of the tests in all cases. Thus, the data substantiates a general susceptibility of Gram-positive bacteria against Ps-CLPs.

**Table 3:** Overview of the number of “hits” compared to the number of tests *n* reported against Gram-positive bacteria per CLP group.

	Syringomycin	Viscosin	Xantholysin	Tolaasin	Syringopeptin
*Bacillus*	4 (*n* = 6)	12 (*n* = 15)	3 (*n* = 3)	11 (*n* = 12)	13 (*n* = 13)
*Clavibacter*		4 (*n* = 4)		4 (*n* = 4)	
*Clostridium*		6 (*n* = 6)			
*Enterococcus*		11 (*n* = 12)			
*Micrococcus*	2 (*n* = 3)	2 (*n* = 4)			
*Propionibacterium*		4 (*n* = 4)			
*Rhodococcus*		2 (*n* = 3)	3 (*n* = 3)	8 (*n* = 9)	
*Staphylococcus*		15 (*n* = 30)			8 (*n* = 12)
*Streptococcus*		8 (*n* = 8)			


As for Gram-negative bacteria, 181 tests were reported against a panel of in total 19 genera (representing 45 species), of which 51 tests were “hits” (28%) (**Supplementary Table 4**). From all the data (**Table 4**), 6 Ps-CLP groups can be identified where the number of tests *n* ≥ 3 on a genus-level, involving 7 genera (**Table 4**), with *Escherichia* and *Pseudomonas* being most extensively tested. The lack of activity originally suggested to exist for Ps-CLPs in the case of Gram-negative bacteria (Nybroe and Sørensen, 2004; Reder-Christ et al., 2012) should be nuanced. Although clearly less successful than against Gram-positive bacteria, the different cell envelope architecture of Gram-negative bacteria does not provide absolute protection from Ps-CLPs. A notable difference with Gram-positive bacteria is the larger selectivity. Indeed, when testing Gram-negative bacteria none of the 30 tests featuring Ps-CLPs from the syringopeptin group were positive. In contrast, 16 of the 21 tests (76%) of xantholysin against Gram-negative bacteria were positive, with potent effect against *Pseudomonas* and *Xanthomonas*, but no effect against *Escherichia*. A more in depth analysis learns that no overlap exists between the collection of species used to test xantholysin and syringopeptin, respectively, which is necessary before a generalization can be made. Interestingly, the tolaasins, which like syringopeptin, are large peptide amphiphiles with positive charge, do show activity against certain Gram-negative species which, again, do not overlap with those of the other two Ps-CLPs. Given the larger selectivity observed here, more data, against identical species, should be collected before any generalization can be made. Indeed, it may well turn out that xantholysins are equally inactive against those used to test the syringopeptin CLPs, whereas the latter may also be active against those exposed to xantholysin. More generally, when considering only those bacteria of which *n* ≥ 3 per CLP group, conclusions can solely be made for a limited number of Gram-negative bacterial genera (**Table 4**). All tested Ps-CLP groups do not exhibit activity against *Citrobacter, Erwina, Escherichia, Proteus*, or *Salmonella* genera. In case of *Pseudomonas* and *Xanthomonas* genera, there appears to be a discrimination depending on the CLP group. Although the xantholysins (*n* = 12) and the tolaasins (*n* = 6) are produced by *Pseudomonas* spp., they do appear to have (some) activity against this bacterium. However, both groups were not tested against the same species. Specifically, the tolaasins are able to inhibit the growth of *P. syringae* and *P. corrugata* whereas the xantholysins possess activity against *P. aeruginosa, P. putida, P. mendocina*, and *P. nitroreducens*. Finally, *Xanthomonas* spp. are not susceptible to syringomycins, but are to xantholysins (*n* = 3) and viscosins (*n* = 16). However, the latter is exclusively due to results collected for WLIP. Therefore, rather than stating that viscosins have activity against Gram-negative bacteria, a more accurate conclusion would be that WLIP, a CLP of the viscosin group, is active against *Xanthomonas* spp.

**Table 4:** Overview of the number of “hits” and the number of tests *n* reported against Gram-negative bacteria per CLP group.

	Syringomycin	Viscosin	Putisolvin	Tolaasin	Xantholysin	Syringopeptin
*Citrobacter*						0 (*n* = 4)
*Erwinia*	1 (*n* = 3)	1 (*n* = 3)		3 (*n* = 10)		
*Escherichia*	1 (*n* = 3)	0 (*n* = 12)		2 (*n* = 12)	0 (*n* = 4)	0 (*n* = 6)
*Proteus*						0 (*n* = 4)
*Pseudomonas*	1 (*n* = 5)	0 (*n* = 21)	0 (*n* = 4)	6 (*n* = 11)	11 (*n* = 12)	0 (*n* = 8)
*Salmonella*		0 (*n* = 5)				0 (*n* = 4)
*Xanthomonas*	1 (*n* = 3)	14 (*n* = 16)			3 (*n* = 3)	


From the above, it is clear that comparison of the reported biological activities of different CLPs in order to assess the effect of certain structural modification is precluded by the fact that collections of microorganisms used in the individual studies are mostly non-overlapping. When attempting to establish a structure-activity relation for the Ps-CLPs, one has been limited to the natural variations that occur.

Recently, the biological activities of different natural CLPs of the viscosin group were assessed, showing that the D/L stereo-inversion that occurs in the viscosin group does not have an impact on the antibacterial activities (Geudens et al., 2017). In contrast, WLIP-(V) and viscosin, containing an ionisable D-Glu2, generally had a lower activity compared to their respective D-Gln2 homologs pseudodesmin A-(V) and viscosinamide A-(V). This finding is thought to be related to an altered membrane-water partitioning.

Alternatively, chemical peptide synthesis can prove a valuable tool to establish a structure-function relation of the Ps-CLPs. For example, using an efficient solid-phase peptide synthesis approach, the enantiomer of pseudodesmin A, a viscosin group member, showed identical activity to the parent compound when confronted with a panel of Gram-positive bacteria (De Vleeschouwer et al., 2014). Thus, a chiral, receptor based interaction is not the main determinant of its biological activity (De Vleeschouwer et al., 2014). Taken together, these findings already provide an initial insight in the working mechanism of the Ps-CLPs, and with the availability of efficient synthesis routes toward Ps-CLPs the generation of chemical structure diversity to further investigate the structure-activity relationship studies can be expected.

## Conclusion

Given the many different biological environments where *Pseudomonas* species play a role, they truly are “multi-talented bacteria” (Keel, 2016) and with exception of *Pseudomonas aeruginosa*, their specialized metabolome constitutes a rich source of amphiphilic CLPs. As a family, Ps-CLPs appear as the equivalent of a “Swiss-Army knife” capable of a wide range of biological and functionally relevant effects, using the same overall molecular blue-print. In addition and even though extensive biological data only exists for a limited few Ps-CLPs, the data of WLIP and tolaasin indicate that this multi-tool character can also be attributed to individual Ps-CLP members. Ps-CLPs thus appear as a rich source of compounds in fundamental and application oriented research. The antifungal activity appears to be the most generally expressed property of Ps-CLP, followed by activity against Gram-positive bacteria. Antagonistic effects against Gram-negative bacteria, while present, are clearly more selective and currently more limited toward specific organisms. In all cases, however, some or considerable tolerance can be found against specific genera. Therefore, generalizations regarding the biological activities of Ps-CLPs should be avoided or presented with the appropriate context.

In spite of the increasingly large body of activity data, the large diversity in the handful of organisms selected for activity assays from one study to another, let alone the diversity in procedures used and concentration ranges tested, limits meaningful comparison of the potency of Ps-CLPs or derivation of structure-activity relationships to a few very specific cases. In our opinion, this causes considerable hindrance for the further exploration and development of Ps-CLPs as biological agents (Geudens et al., 2018). This could be partly addressed by defining a common series of organisms, reference strains (i.e., ATCC, NCPPB, BCCM, etc.) cancer-cell lines etc., as well as assaying procedures and practices that could be made available to characterize newly isolated Ps-CLPs, irrespective of the particular interest of the investigating research group. The data could then be used to annotate existing databases (e.g., NORINE) or to develop new ones (Geudens et al., 2018). In this way, identification of a newly isolated compound as an already described Ps-CLP would immediately also provide functional information, while the availability of uniform testing protocols would immediately allow to relate the functional repertoire of a novel, previously undescribed compound to the existing library of information. This is particularly pertinent given the recent development of metabolite indexing approaches using mass-spectrometry that promise to accelerate the discovery of new metabolites compared to the usual one-molecule–one-microbe at a time approach, as was recently demonstrated for Ps-CLPs (Nguyen et al., 2016). Only by investing in functional annotation of metabolites or groups of metabolites – such as Ps-CLPs – using *a priori* agreed upon assays, will it be possible to realize the full scientific potential that they may hold for science and society.

## Author Contributions

NG collected and organized all relevant literature data and information to write the outline of this review, wrote the initial manuscript, and took care of all figure and tabular data. Both authors shared equal credit in the iterative process of producing the final outline, all data interpretation, and the discussion.

## Conflict of Interest Statement

The authors declare that the research was conducted in the absence of any commercial or financial relationships that could be construed as a potential conflict of interest.
