# Supplementary material for: Cyclic Lipodepsipeptides From Pseudomonas spp. – Biological Swiss-Army Knives
Source: Front Microbiol. 2018 Aug 14;9:1867. doi: 10.3389/fmicb.2018.01867 (PMC6104475; doi:10.3389/fmicb.2018.01867)
Supplement: Supplementary file 1 [file Data_Sheet_1.pdf]

Supplementary Material

Cyclic Lipodepsipeptides from Pseudomonas spp. – Biological and Functional Swiss-Army Knives

Niels Geudens[1,*], José C. Martins[1,*]

*** Correspondence:**
Dr. Niels Geudens,
niels.geudens@ugent.be

Prof. Dr. José C. Martins,
jose.martins@ugent.be

1 Supplementary Data

In order to assess the biological activities of *Pseudomonas* spp. CLPs, an extensive Excel-table was drafted whereby the many different biological activities of *Pseudomonas* spp. CLPs are listed.

TABLE S1 | OVERVIEW OF THE NUMBER OF BIOLOGICAL TESTS PERFORMED FOR DIFFERENT Ps-CLPs ON THE DIFFERENT CATEGORIES OF MICRO-ORGANISMS. BLANKS INDICATE THAT NO TESTS WERE REPORTED.

		# tests	All targets		Gram-positive		Gram-negative		Myco-bacteria		Fungi		Others	
			Hits	Misses	Hits	Misses	Hits	Misses	Hits	Misses	Hits	Misses	Hits	Misses
	**TOTAL**	**775**	**506**	**268**	**141**	**39**	**51**	**130**	**19**	**3**	**248**	**58**	**48**	**38**
**VISCOSIN**	Total	298	165	133	68	26	15	57	8	2	61	34	13	14
	Viscosin	27	16	11	8	7	0	2	3	0	2	2	3	0
	Viscosinamide	28	25	3	13	1			1	0	9	2	2	0
	Pseudodesmin A	26	17	9	16	1	0	4	1	0	0	4		
	Pseudodesmin B	11	5	6	5	0	0	3			0	3		
	WLIP	160	69	91	18	14	15	40	1	2	30	23	5	12
	Massetolide A	20	15	5	0	1	0	2	2	0	12	0	1	2
	Pseudophomin A	13	9	4	4	1	0	3			4	0	1	0
	Pseudophomin B	13	9	4	4	1	0	3			4	0	1	0
**ORFAMIDE**	Total	21	17	4							16	3	1	1
	Orfamide A	11	7	4							6	3	1	1
	Orfamide B	6	6	0							6	0		
	Orfamide G	4	4	0							4	0		
	PPZPMs													
**AMPHISIN**	Total	20	8	11	0	4	0	2	0	1	4	3	4	1
	Amphisin	2	2	0							2	0		
	Arthrofactin	1	1	0									1	0
	Lokisin	2	2	0							2	0		
	Anikasin	14	3	11	0	4	0	2	0	1	0	3	3	1
	Tensin	1	1	0							1	0		
**TOLAASIN**	Total	145	113	32	27	2	14	22			68	8	4	
	Tolaasin I	109	94	15	17	0	14	10			59	5	4	0
	Tolaasin II	6	4	2	2	0	0	2			2	0		
	Tolaasin A	6	4	2	2	0	0	2			2	0		
	Tolaasin B	6	3	3	2	0	0	2			1	1		
	Tolaasin C	6	1	5	0	2	0	2			1	1		
	Tolaasin D	6	4	2	2	0	0	2			2	0		
	Tolaasin E	6	3	3	2	0	0	2			1	1		
**SYRINGOMYCIN**	Total	95	78	17	10	3	5	10	1	0	48	3	14	1
	Syringomycin E	42	34	7	6	1	5	5	1	0	17	0	6	1
	Cormycin A	6	6	0	1	0					1	0	4	0
	Pseudomycin A	5	5	0							5	0		
	Pseudomycin B	6	4	2							4	2		
	3'-epi PSB	3	2	1							2	1		
	C18-PSB	3	3	0							3	0		
	C20-PSB	4	4	0							4	0		
	Thanamycin	7	7	0	1	0					6	0		
	Syringotoxin	20	13	7	2	2	0	5			7	0	4	0
**SYRINGOPEPTIN**	Total	108	67	41	24	4	0	30	9	0	31	7	3	0
	Corpeptin A	2	1	1	1	0					0	1		
	Corpeptin B	2	1	1	1	0					0	1		
	SP508A	18	12	6	4	1	0	5	2	0	6	0		
	SP508B	18	12	6	4	1	0	5	2	0	6	0		
	SP22A	34	23	11	7	1	0	10	3	0	11	0	2	0
	SP22B	18	12	6	4	1	0	5	2	0	6	0		
	SP22PhvA	2	0	2							0	2		
	SP25A	14	6	8	3	0	0	5			2	3	1	0
	MDN-0066	1	1	0									1	0
	Pseudofactin	4	4	0	1	0	1	0					2	0
**XANTHOLYSIN**	Total	70	46	24	10		16	5	1	0	13	0	6	19
	Xantholysin A	41	34	7	7	0	10	3	1	0	13	0	3	4
	Xantholysin B	6	1	5									1	5
	Xantholysin C	16	9	7	3	0	6	2					0	5
	Xantholysin D	5	0	5									0	5
	MA026	2	2	0									2	0
**PUTI-SOLVIN**	Total	6		6			0	4					0	2
	Putisolvin I	3	0	3			0	2					0	1
	Putisolvin II	3	0	3			0	2					0	1

TABLE S2 | Overview of the number of 'hits' and the number of tests n reported against fungi per CLP group. Fungal genera for which only limited tests are performed (n < 3) are indicated by "T".

	Viscosin	Orfamide	Amphisin	Tolaasin	Syringomycin	Syringopeptin	Xantholysin
Agaricus	T			4 (n= 4)			
Alternaria	T			T			T
Armillaria	T			T			
Ascochyta							T
Aspergillus	0 (n= 4)				7 (n= 10)		
Bipolaris					T		
Botrytis	T			T	3 (n= 3)	T	T
Candida	0 (n= 10)	T	T	2 (n= 4)	7 (n= 7)	8 (n= 8)	
Chlamydomonas					T		
Colletotrichum							T
Coprinus				T			
Cryptococcus	0 (n= 4)			T	7 (n= 7)		
Flammulina				T			
Fusarium	4 (n= 6)			6 (n= 6)	T		5 (n= 5)
Geotrichum	T			T	6 (n= 6)	3 (n= 6)	
Gloeosporium							T
Heterobasidion	T			T			
Lentinus	T			3 (n= 3)			
Magnaporthe		3 (n= 3)					
Malassezia	T			T			
Mucor					T		
Nectria							T
Penicillium			T		T		
Phoma	3 (n= 3)			T			
Phytophthora	4 (n= 8)	4 (n= 4)	T	4 (n= 4)	T	T	
Pleurotus	4 (n= 5)			9 (n= 9)			
Pyrenophora							T
Pythium	14 (n= 14)	3 (n= 4)	T		T	T	
Rhizoctonia	8 (n= 8)	5 (n= 6)	T	9 (n= 9)	T	T	T
Rhodotorula	T			7 (n= 10)	6 (n= 6)	19 (n= 23)	
Saccharomyces	T						
Saprolegnia	T						
Sclerotinia	6 (n= 6)			4 (n= 4)			
Sclerotium	T			T			
Septoria				T			
Trichoderma	T			T			
Trichophyton	T						
Verticillium	T			T			
Volvariella				T			

TABLE S3: OVERVIEW OF THE NUMBER OF 'HITS' AND THE NUMBER OF TESTS N REPORTED AGAINST GRAM-POSITIVE BACTERIA PER CLP GROUP. GRAM-POSITIVE GENERA FOR WHICH ONLY LIMITED TESTS ARE PERFORMED (N < 3) ARE INDICATED BY "T".

	**VISCOSIN**	**ORFAMIDE**	**AMPHISIN**	**TOLAASIN**	**SYRINGOMYCIN**	**SYRINGOPEPTIN**	**PSEUDOFACTIN**	**XANTHOLYSIN**
ARTHROBACTER	T	T	T		T			
BACILLUS	12 (n= 17)	T	0 (n= 3)	11 (n= 12)	4 (n= 8)	13 (n= 13)		3 (n= 3)
CLAVIBACTER	4 (n= 7)	0 (n= 3)	3 (n= 3)	4 (n= 4)	1 (n= 4)			
CLOSTRIDIUM	6 (n= 6)							
CORYNEBACTERIUM	1 (n= 4)	0 (n= 3)	3 (n= 3)	T	1 (n= 4)			
CURTOBACTERIUM	T			T				
ENTEROCOCCUS	11 (n= 13)	T	T		T			
GEOBACILLUS	T							
LACTOBACILLUS	T							
LISTERIA	0 (n= 3)	T	T		T			
LYSINIBACILLUS								T
MICROCOCCUS	2 (n= 5)	T	T	T	2 (n= 4)	T		
PROPIONBACTERIUM	4 (n= 4)							
RHODOCOCCUS	2 (n= 3)			8 (n= 9)	T	T		3 (n= 3)
STAPHYLOCOCCUS	15 (n= 36)	0 (n= 6)	0 (n= 8)	T	0 (n= 6)	8 (n= 12)	T	
STREPTOCOCCUS	8 (n= 8)							
STREPTOMYCES					T			T

TABLE S4 | OVERVIEW OF THE NUMBER OF 'HITS' AND THE NUMBER OF TESTS N REPORTED AGAINST GRAM-NEGATIVE BACTERIA PER CLP GROUP. GRAM-NEGATIVE GENERA FOR WHICH ONLY LIMITED TESTS ARE PERFORMED (N < 3) ARE INDICATED BY "T".

	**VISCOSIN**	**ORFAMIDE**	**AMPHISIN**	**TOLAASIN**	**SYRINGOMYCIN**	**SYRINGOPEPTIN**	**PSEUDOFACTIN**	**XANTHOLYSIN**	**PUTISOLVIN**
AEROMONAS	T								
AGROBACTERIUM	T			T	T				
AZOSPIRILLUM	T								
BORDETELLA	T								
BURKHOLDERIA	T							T	
CITROBACTER	T	T	T		T	0 (n= 4)			
ENTEROBACTER	T								
ERWINIA	1 (n= 3)			3 (n= 10)	1 (n= 3)	T			
ESCHERICHIA	0 (n= 14)	T	0 (n= 3)	2 (n= 12)	1 (n= 5)	0 (n= 6)		0 (n= 4)	
KLEBSIELLA	T	T	T		T				
PROTEUS	T					0 (n= 4)	T		
PSEUDOMONAS	0 (n= 22)	T	T	6 (n= 11)	1 (n= 6)	0 (n= 8)		11 (n= 12)	0 (n= 4)
SALMONELLA	0 (n= 5)					0 (n= 4)			
SERRATIA	T	T	T		T				
SHIGELLA	T								
SPHINGOMONAS	T							T	
VARIOVORAX	T								
XANTHOMONAS	14 (n= 16)			T	1 (n= 3)	T		3 (n= 3)	
YERSINIA	T								

2 Supplementary Figures

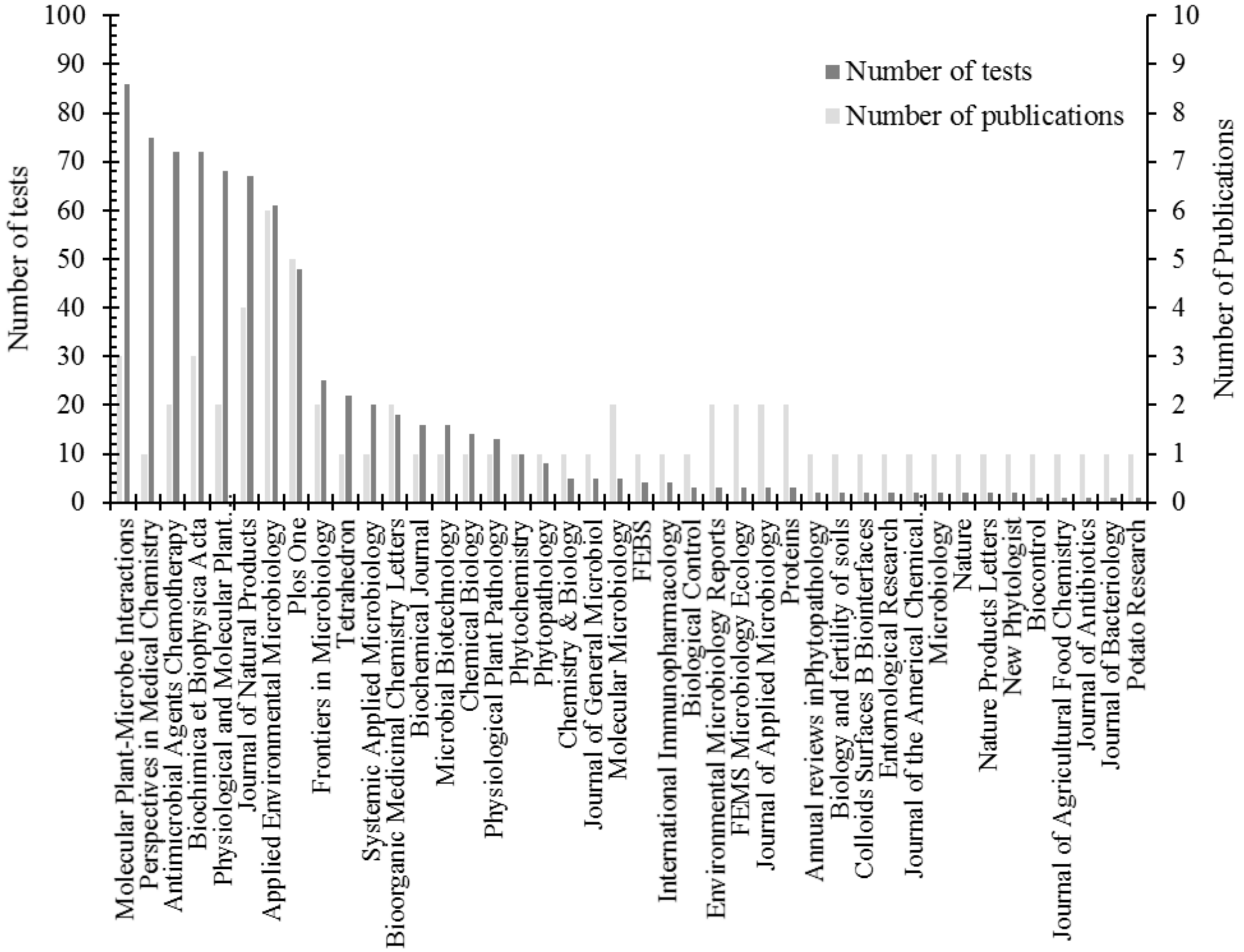

Supplementary Figure 1 | Number of publications and number of tests reviewed here. It is noteworthy that 561 (72.5%) of all biological tests were published in 8 different journals.

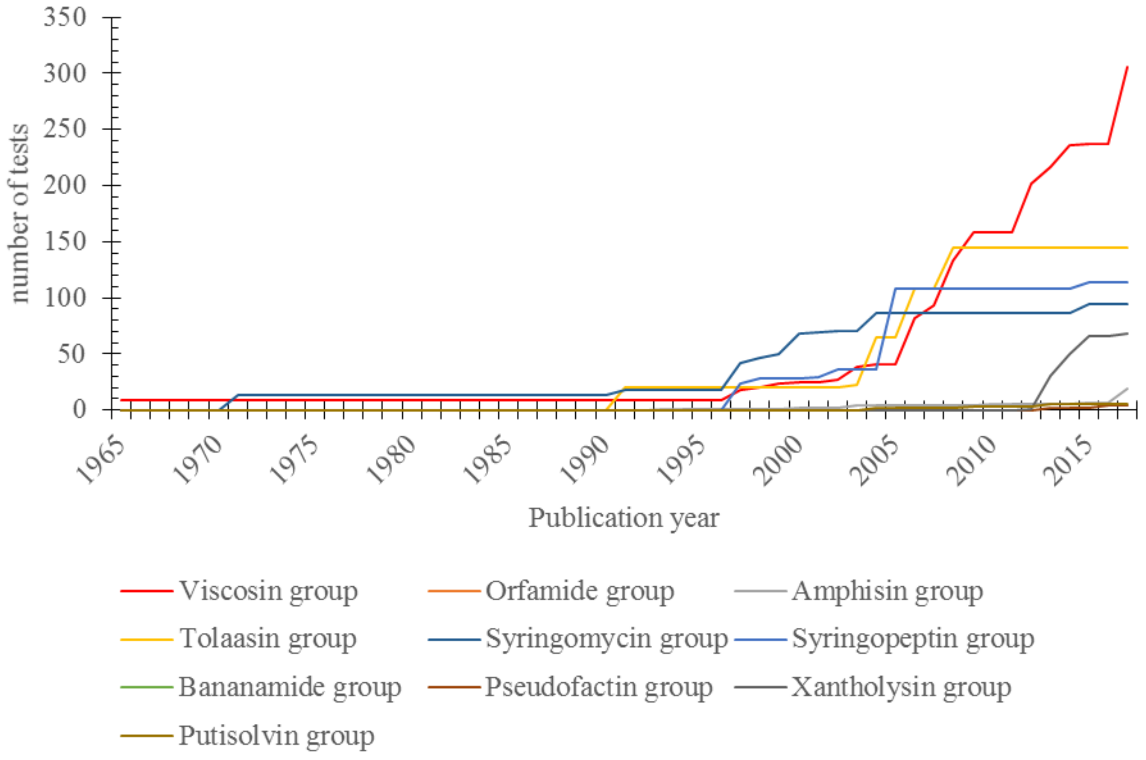

Supplementary Figure 2 | Number of tests reported as a function of publication year.

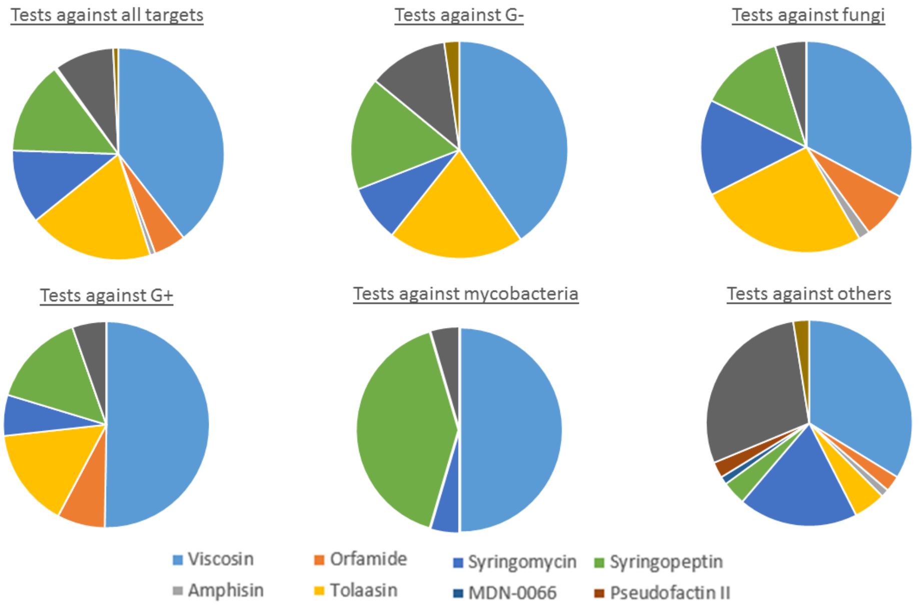

Supplementary Figure 3 | Distribution of all biological tests according to CLP group classification.

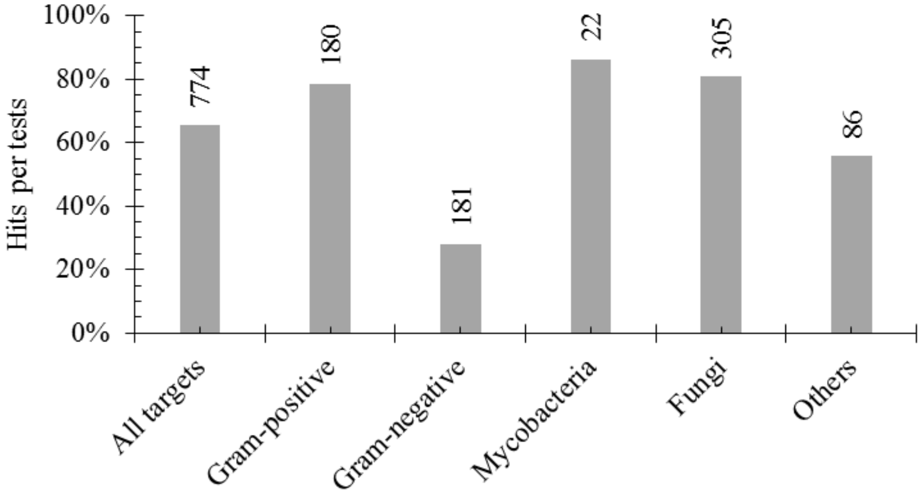

Supplementary Figure 4 | Number of hits per tests for all CLP groups per micro-organism category.
